# Prescriptions

**Published:** 1896-10

**Authors:** 


PHARYNGITIS.

℞ Iodol........................ʒj

Spts. vini rectificati..........℥ij

Glycerinæ.............. . ℥iv. ʒij

M. Sig.: Apply with a brush or as a coarse spray.—*Wolfenden Annual Univ. Med. Sci.*

URAEMIA.

℞ Pulv. scillæ....................
Pulv. scammonii...............
Pulv. digitalis......aa gr. xv.
M. Ft. massa et in pil. no. xx. div.
Sig.: Four to six pills daily, for five or six days.—*Lancereaux.*

WHOOPING-COUGH.

℞ Sodii benzoatis..............℈iv

Aquæ menthæ pip..........

Aquæ destillatæ..........aa ʒx

Syr. aurantii ... ...........ʒij

M. Sig.: A dessertspoonful every hour or two.—*Letzerich.*

WOUNDS.

℞ Acidi tannici...............℥iiss

Alcoholis absoluti..........℥ss

Ætheris .. ........... ....℥iiss

Collodii .. .... .........℥xij

M. Sig.: "Styptic colloid."

ABSCESSES.

℞ Iodiformi............ʒiiss–ʒv.
Ætheris.......................℥vj.
M. Inject three to five ounces after aspirating the abscess. (*In cold or tubercular abscess.*)—*Mosetig-Moorhof, Annual Univ. Med. Sci.*

